# A Serious Game to Improve Cognitive Functions in Schizophrenia: A Pilot Study

**DOI:** 10.3389/fpsyt.2016.00064

**Published:** 2016-04-20

**Authors:** Isabelle Amado, Lindsay Brénugat-Herné, Eric Orriols, Colombe Desombre, Maxine Dos Santos, Zelda Prost, Marie-Odile Krebs, Pascale Piolino

**Affiliations:** ^1^Sainte Anne Hospital, Paris, France; ^2^Reference Center for Cognitive Remediation and Psychosocial Rehabilitation, INSERM U894, Sainte Anne Hospital, Paris, France; ^3^Center for Psychiatry and Neuroscience, INSERM U894, Paris Descartes University, Paris, France; ^4^Laboratory for Memory and Cognition, Sorbonne Paris Cité, Paris, France; ^5^Institut Universitaire de France (IUF), Paris, France

**Keywords:** cognition, schizophrenia, virtual reality, serious game

## Abstract

Cognitive deficits in schizophrenia impair everyday functioning and instrumental daily living activities. These disabilities can be partly responsible for chronicity and institutionalization. We present here a virtual reality (VR) tool in which patients with schizophrenia performed a virtual game in an imaginary town during a 3-month program. In a pilot study, seven patients with schizophrenia (DSM-5), institutionalized for many years, attended weekly 1-h-and-a-half sessions organized by two clinicians. During the first sessions, they listed together the difficulties they experienced in everyday organization and planning. After being familiarized with the joystick and the VR environment, they navigated in the town, and planned actions that were difficult for them to carry out in their usual life (e.g., shopping, memorizing the way to the supermarket or being on time at a meeting point). They had to look for alternative routes and practice a switch from a 2D Map to the 3D Map. They also gathered their efforts to share strategies for each action, or discussed the action plan they could generate to solve concrete problems. The pre/post-neuropsychological evaluations showed attention, working memory, prospective, and retrospective memory benefits, but no improvement in planning as assessed by the Zoo map test and the action program subtest of Behavioral Assessment of the Dysexecutive Syndrome. Patients also clinically and functionally improved, gaining autonomy. Pragmatically, they reported a strong energy to elaborate concrete plans to search for jobs, or return to activities in the community. Qualitative assessments showed a benefit in sparing time, planning better, enriched relatedness, and better management of their housework. This VR game opens avenue to rehabilitation for patients with schizophrenia experiencing chronicity in their life, less attendance in daycare units, and a better community living. This program might reduce neurocognitive difficulties and might evolve into a true method for cognitive remediation (trial n° 2011-A00988-33).

## Introduction

Cognition impairments in schizophrenia are found in multiple domains, with direct implications in everyday functioning (1). These difficulties limit the patients’ access to full time employment, independence in their residential living, and social outcomes (2). Green et al. (3) revealed associations between specific neurocognitive constructs and functional outcome. Josman et al. (4) reported a link between deficits in executive functioning and ability to perform daily activities in subjects with schizophrenia using the instrumental activities of daily living scale (5). Actually, psychosocial therapies and more specifically cognitive remediation are necessary to complete pharmacological treatments to alleviate cognitive deficits and improve patients’ everyday functioning.

Although neuropsychological tests provide important information about cognitive disabilities, they generally have low ecological validity and, therefore, have limited ability to predict functioning in daily activities (6). The term “ecological validity” is central in the area of assessment of crucial functions, such as executive functioning, as these functions are fully associated with real-life complex situations (e.g., shopping, preparing a meal, or medication adherence) that require planning, organization, and structuring (7). Very recently, the apprehension of everyday functioning has been enriched with the development of computerized assessment of functional capacities using virtual reality (VR). VR is supposed to mimic the real world in an immersive and potentially remotely deliverable environment. In psychology, VR provides an immersion in a safe, non-stressful, and ecologically valid environment, while maintaining strict experimental control over stimulus delivery and measurement (8). Moreover, VR enables users to be engaged in simulated, interactive environments that are similar to real-world objects and events (9). These environments are multimodal and offer the opportunity to record all modalities of cognitive and behavioral activities (10–12). In the executive functioning domain, studies using VR reported that the immersion in that world and in a meaningful context facilitates performance (13).

In the psychosis domain, Kurtz et al. (14) used a virtual apartment to evaluate the medication monitoring in patients with schizophrenia versus that of controls: patients made more errors in the number of pills taken, were less accurate at taking medication, and were less attentive to the hour the pills had to be taken. These defects reported in VR were in agreement with real-life reports, through a classification of adherence versus non-adherence in medication management skills. Josman et al. (4) compared planning abilities in patients with schizophrenia versus controls with a shopping task, using the Virtual Action Plan supermarket (15), while examining the correlations with the Behavioral Assessment of the Dysexecutive Syndrome [BADS (16)]. Patients had more difficulties than controls, specifically to perform the different actions, such as buying virtually all the items on the shopping list. Zawadzki et al. (12) evaluated visuo-spatial organization and spatial orientation during a virtual navigation in a realistic city. Individuals with schizophrenia had difficulties in route-finding within the virtual city. They were also more likely not to notice the target during passive viewing, not to find novel shortcuts to targets, and more likely to become lost and fail completely in finding the target. Scores were negatively correlated to neurocognition as well as to the Quality of Life Scale and to psychosocial functioning. Lastly, Ruse et al. (17) proposed the virtual reality functional capacity assessment tool (VRFCAT) to evaluate performance in everyday life in an ecological manner, with different versions of short scenarios that include navigating in a kitchen, planning a trip to the grocery store, and therefore taking a bus, organizing all the steps to go shopping. Patients with schizophrenia compared to controls showed difficulties in several items. Also, strong correlations were found with slowing of information processing and working memory evaluated with the Matrix Consensus Cognitive Battery.

However, in the neurocognitive domain, if VR is mainly used to evaluate cognitive abilities in an ecological manner, no program exists to improve executive functioning in a VR environment, supposed to mimic the real world.

Our aim was to test a new method to improve cognitive abilities, especially memory, executive functioning, and planning in patients with schizophrenia, using a virtual city. Therefore, we mainly focused our pre–post-neuropsychological evaluations on retrospective and prospective memory, working memory, learning, and planning abilities. A total of twelve 1-h-and-a-half weekly sessions during a 3-month program were delivered in groups of a maximum of six patients. As VR provides an immersive non-stressful environment, we wanted to propose a VR program to patients with schizophrenia who were institutionalized, with long-time use of daycare programs of therapeutic activities. These patients, because of their very ritualized way of living, were considered as more chronic than other patients and less susceptible to accept spontaneously a direct access to rehabilitation perspectives, such as job employment or professional training. Also, it was a challenge for our unit, because institutionalized patients are difficult to drive to innovative therapies, and to orient toward external activities that take place in the real world. This very preliminary open study was a clinical trial with raters who were not blind to assessments and to treatments, with no randomization, and no control arm.

Our hypothesis stated that a VR environment improves patients’ cognitive abilities, mainly in prospective memory and planning, with a clinical benefit and a benefit in social functioning, quality of life and self-esteem, assessed in a pre/post comparison before and after the VR program.

## Materials and Methods

### Population

Ten outpatients (in two different groups) meeting Diagnostic and Statistical Manual of Mental Disorders, Fifth Edition (DSM-5) (18) criteria for schizophrenia or schizoaffective disorders were recruited. Patients had been institutionalized for several years, coming several times a week to a daycare activity center (Centre d’activité thérapeutique à temps partiel, Rue Mathurin Régnier, 75015 Paris). Two patients dropped out after the first two sessions. Exclusion criteria included auditory or visual impairment, mental retardation (IQ < 70), traumatic brain injury, presence or history of neurological illness, bad understanding of French or instructions, and/or criteria met for concurrent substance abuse or dependence. All participants provided written informed consent, and all procedures met institutional ethical approval, following the declaration of Helsinki (trial n° 2011-A00988-33). Finally, although eight participants were able to complete the clinical assessments, only seven of them performed the pre/post neuropsychological tests, because one patient refused to be evaluated after the program, and only six participants performed VR game assessment due to a technical problem during the preprogram evaluation.

### Procedure

This study took place in our Reference Center for Cognitive Remediation and Rehabilitation (C3RP – Paris Descartes University – Sainte Anne Hospital – Paris), in a collaborative partnership with a specialized unit for memory assessment via VR (Laboratory of Memory and Cognition, LMC – Paris Descartes University).

#### The Virtual Town

The virtual town was developed and created by the LMC unit. It consisted of virtual urban environments built with LMC software EditoMem [based on3DVIA Virtools Dev 5.0 software (3dVIA Virtools)] allowing us to create virtual environments inspired by photos of Paris and game scenarios.

**Figure 1:**
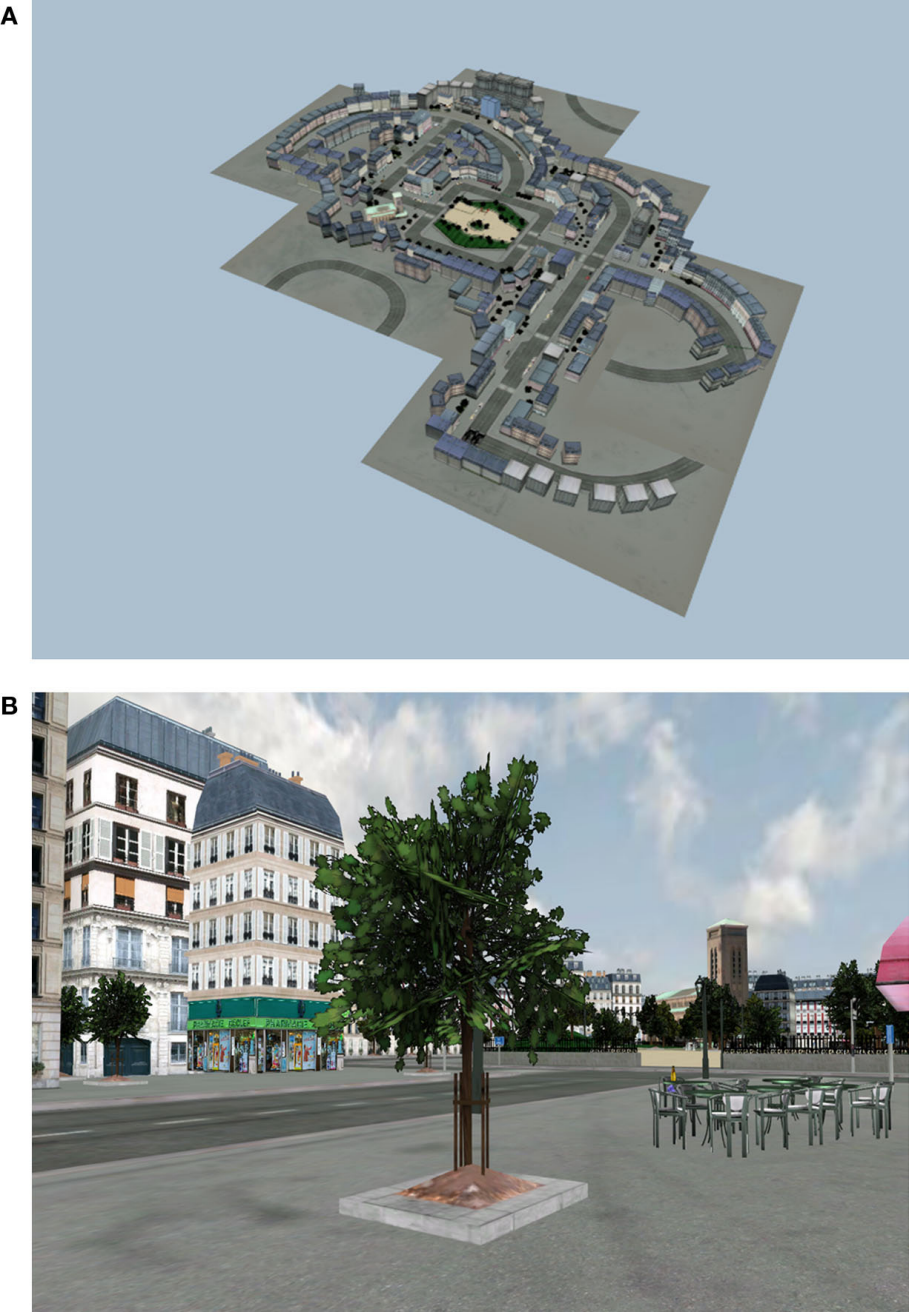
**The urban environment created by the LMC unit**. **(A)** Map of the virtual 3D town. **(B)** View of a street in the virtual town.

These urban environments have been previously used in healthy individuals and patients to assess episodic, spatial, retrospective, and prospective memories as well as working memory [e.g., Ref. (19–23)]. In the present virtual town, the main elements were located so that they could be easily detected (i.e., in front of the subjects, in a corner of a street, or quite salient on the side of the road). Patients had to navigate as pedestrians in a virtual 3D town, using a joystick (simulation software: Simulamem), along several avenues and roads (see Figures 1A,B).

**Figure 2:**
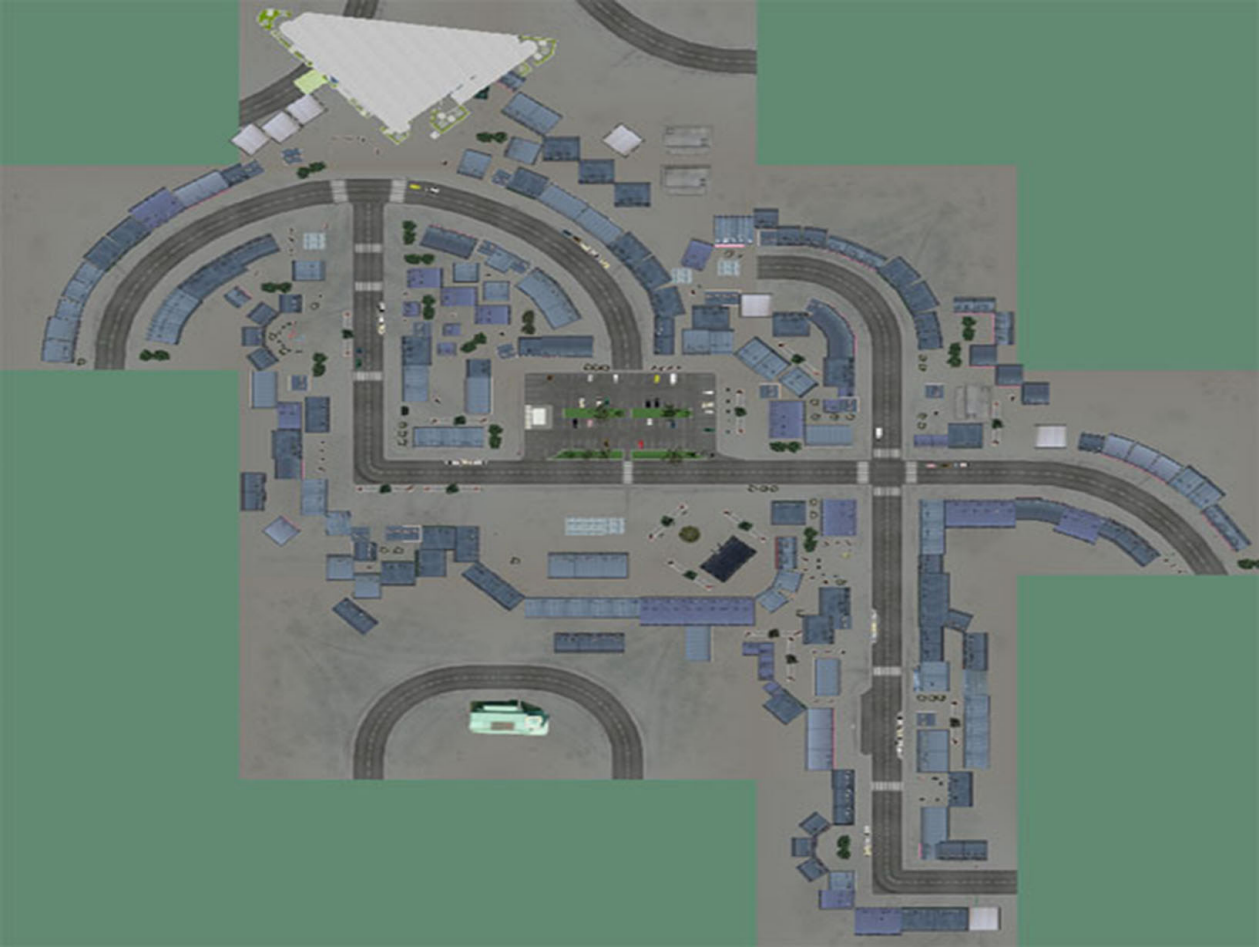
**The map of the virtual environment given as a reference to the participants**.

Several specific elements are located in the town, such as a bank and several shops offering a large diversity for shopping (bakery, grocery, a supermarket). Hobbies are also represented with a cinema and a “mediatheque,” which is a typical French structure where you can freely borrow various types of media. Centers for healthcare also exist with a pharmacy, a dentist practice house and a laboratory for blood analysis. Cafes, restaurants, squares, and a public garden are represented. These different places could be memorized and used as memory cues to orient the navigation, as visual landmarks, or could be a theme for discussion (e.g., what do we have to do in case of a fire beginning in front of you? what are the hypotheses if you come to see a friend and a truck with boxes, which look as a moving out, is located is front of his/her building?). Also, participants, while playing during the VR game, received their own personal 2D map of the virtual town, which they had to complete with street names and mark city’s symbolic places. This map could always be referred, while navigating, to help orientation and train switching from 2D to 3D virtual spaces (see Figure 2). This VR environment was presented in a quiet room with a computer (15.6″), allowing good visibility for the whole group.

#### Description of a Session

The whole program includes 12 interactive 90-min weekly sessions. Two clinicians, a psychologist and an occupational therapist (ZP/LB-CD) conduct the group sessions. During 60 min, one after the other, the participants have to navigate in the VR town, sharing a joystick. While one of the members in the group navigates, the others are helping him, in an interactive and collective effort. Participants have to find their way (involving, therefore, attention and visuo-spatial organization), to memorize their itinerary (which involves memory for details and topographic memory abilities), to plan different actions (encouraging planning ability, flexibility, executive functions, as well as prospective memory), depending on the instruction they have to follow. After 1 h of navigation and several participants navigating in the town, all the participants in the group discuss during the last 20 min of the session about their actions and the possible transfer to everyday life they can imagine. At the end of each session, a task to perform at home is given, directly linked to the theme of the session (trying to reorganize their administrative papers, organizing a party with friends or family members for the Epiphany, etc.). Two different versions of the virtual town have been used, successively versions 1 and 2 during the 12 sessions for the first program (6 sessions for Version 1 and 4 sessions for Version 2). During the two program sessions, we improved and enriched the city, adding more landmarks. Indeed, we added several bakeries, butcher’s shops, and drugstores. We also added a travel agency, two cinemas, a theater, more restaurants and coffee shops, a dairy store, and a florist. Thereby, for the second program, we used a version that could cumulate the different locations existing in Version 1 and the different locations existing in Version 2 for the 12 sessions.

Procedure of the different sessions and thematic contents:

- Sessions 1–4: Familiarization. How to navigate with the joystick? Orientation in a 2D environment; shifting from map to 3D on the screen. Preliminary interactive discussions: everyday life examples that could be sources of emotional distress, problems encountered in everyday life, planning difficulties but also daily activities for which participants have no difficulties. The latter point is to identify the areas where there could be sources of solutions for the group. Encouraging the strategies found by the group. Discovering the VR town on the screen: paying attention to the names of streets, shops, and supermarkets. Categorizing the shops; reporting them on the 2D map. Also, each participant selected a virtual personal address for his/her home in one of the virtual streets. Clinicians encouraged them to imagine their apartment according to their aspirations and preferences. They also asked participants to explain what determined their choices of a particular place.
- Sessions 5–10: Planning and navigation. Discussion around personal strategies to navigate efficiently. Then, on the basis of the difficulties evoked by the groups, different scripts were prepared by the two clinicians: e.g., explaining your route to a pedestrian, trying different routes for the same goal and taking them afterwards. The action scripts tried to mimic as best as possible the everyday life, giving opportunities to participants to relate personal events or difficulties when they navigated (standing in line and eventual distress they could perceive, planning important things in advance, etc.).
- Session 11: Route-finding using the map of the Parisian subway. Navigating from each home address to the big railway station and enunciating all the different steps.
- Session 12: Summary of the entire program. Remarks and comments from the group. Satisfaction questionnaire to encourage criticism and suggest evolution of the program.

#### Clinical and Neuropsychological Evaluations

Two assessments were done, at W0 before the VR program, and W12 after the end of the last session. For each assessment, there was one clinical interview done by a psychiatrist rater who was not blind for the assessments (Isabelle Amado), and the neuropsychological evaluations done by a neuropsychologist, also not blind to the assessments (Lindsay Brénugat-Herné and Zelda Prost) and also involved in the management of the VR sessions. The interviews and the assessments at W12 also collected the subjective opinions of the participants concerning the program and the subjective benefits or harm they perceived.

##### Clinical Scales

– Brief Psychiatric Rating Scale [BPRS (24)] provides an overview of the clinical symptomatology.
– Global assessment functioning scale (GAF) (25). This scale gives a score between 0 and 90, and provides both a global evaluation of the symptomatology and an overview of the functional level of the participant.

###### Psychosocial Scales and Questionnaires

– We assessed the social autonomy of patients with a French scale: the Social Autonomy Scale (EAS) (26). This scale includes five dimensions: personal care, monitoring of everyday life, financial control and relationships with the environment, affective life, and social relatedness.
– The Schizophrenia questionnaire for Quality of life [S-QOL (27)]: scale with 41 items assessing eight dimensions. Each of the items must be scored on a five-point item scale. The different dimensions included psychological well-being, self-esteem, family relationships, relationships with friends, resilience, physical well-being, and autonomy.
– The self-esteem rating scale [SERS-(28), French translation (29)]. This 20-item questionnaire includes a positive evaluation of SERS (P-SERS) and a negative one (N-SERS).
– The Insight dimension was assessed with a Self-report Insight Scale, the Birchwood Insight questionnaire [BIS-(30)].

##### Neuropsychological Evaluation

– Attention, visual scanning abilities, and speed processing: D2 cancellation test (31). The scores considered in the study: D2-KL represents the number of targets correctly canceled; D2-GZ represents the quantitative performance index, that is to say the total number of characters treated; D2-F% represents the quality of the treatment of the information, that is to say, the percentage of errors; D2-GZ-F represents the corrected quantitative performance. Wechsler Adult Intelligence Scale 3rd edition [WAIS-III (32)] – Code subtest: participants have to substitute a number by a symbol as quickly as possible, while the code of the different numbers is noticed in the first line of the sheet. The standard score was considered. This subtest assesses attention and cognitive speed processing – Copy-Code subtest: participants have to copy the symbol appearing in the compartment of the top as quick as possible. The number of compartments completed within the allotted time was considered. This subtest assessed motor speed processing.
– Verbal and visual working memory: respectively, the digit span subtests of the WAIS-III (32) and visuo-spatial span subtests of the clinical memory scale for adults 3rd edition (33) have been selected. For the digit span and the visuo-spatial span, we considered forward and backward span scores and the global standard score.
– Verbal learning: Grober and Buschke verbal learning test (34). The participant had to memorize by three times a list of 16 verbal items. Immediate recall, sum of Free and cued immediate 3 recalls, delayed free and cued recalls, and recognition were considered. Form A of this test was administered at W0 and form B at W12.
– Executive functioning: Zoo map test and action program subtests of the Battery for assessment of dysexecutive syndrome [BADS (16)]. Scores of sequence, time of planning, total time of execution, and the number of errors for both versions 1 and 2 of the tests were considered.
– Visuo-spatial abilities: Rey–Osterrieth Complex Figure Test (RCFT) (35, 36). Total scores and time for copying this complex figure were considered.
– Retrospective and prospective memory virtual test (19, 20): after being familiarized with the device, the subjects had to navigate *via* a joystick two times in the same urban environment [e.g., a post office, a car accident, a cafeteria terrace, a park, a disk jockey (with break-dancers), a bus station] in which they had to pick up a friend at the train station (indicated by panels illustrating a train and a directional arrow). In the first navigation, named the retrospective memory test, subjects were asked to pay attention to the elements/events they encountered along the way and their spatiotemporal contexts. Directly after this navigation phase, forced-choice visual recognition tests were carried out to assess the performance related to retrospective components of episodic memory taking into account the feature binding skills (i.e., capacity to recall factual elements associated with their spatiotemporal contexts). The number of correct recognitions (yes-no response) was recorded regarding (1) factual elements (“what,” e.g., was there this white delivery van?), (2) spatial references with questions for “egocentric where” (e.g., did you see the post office on your right?), and (3) temporal order (“when,” e.g., Are these three images in the correct temporal order?).

Before the second navigation to test the prospective memory performance, and approximately 20 min after the first navigation, subjects were instructed to encode a list of 16 action–intentions from verbal cues and figures presented on a screen: 12 event-based with incongruent or congruent cue actions (e.g., “at the bus stop, I should sing a song,” “ take money, at the post office”) and 4 time-based actions (e.g., “call a friend after 2 min”). The encoding was rehearsed three times to allow subjects to store up clue actions. If at the third rehearsal, subjects obtained a recall of action–intentions below 8, the prospective task was not administered. Being again immersed in the virtual town, they were requested to stop walking at the appropriate time or place, and tell the experimenter what they had to do. We recorded the total recall of intentions at the third rehearsal before navigation (learning score), the number of correct stop at the appropriate scene or time and the number of correct related action. Then, we asked the participants to recall the list of intentions after navigation [for similar scoring method, see Debarnot et al. (20)].

#### Statistical Analyses

Clinical assessments as well as neuropsychological performance and psychosocial evaluations between W0 and W12 were assessed with a unilateral non-parametric Wilcoxon test (Statistica V.10). For each score, mean and SDs were expressed.

## Results

### Socio-Demographical Description of the Participants

Participants were seven men and one woman. Their mean age was 38.6 (12.1), years of education was 12 (1.1). Duration of disease was 13.2 (7.6). All patients were treated, the mean and SD values for chlorpromazine equivalents were162.7 (150.1); for details of treatment, see Table 1.

**Table 1:** **Details for the different treatments for the eight subjects participating to the entire virtual reality program**.

	P1	P2	P3	P4	P5	P6	P7	P8
Treatment	Clozapin Aripiprazole Chlorpromazine Fluoxetine Bromazepam	Quetiapin Venlafaxine Alprazolam	Aripiprazole Zopiclone	Olanzapine Venlafaxine	Aripiprazole Diazepam	Aripriprazole	Clozapin Levothyroxine sodique Fenofibrate Promegestone	Aripriprazole
Chlorpromazine equivalent (mg)	417.8	122.8	39.8	26.6	79.7	119.5	416.10	79.7

BPRS was also clinically assessed (see Table 2).

**Table 2:** **Clinical and psychosocial evaluations**.

	BPRS (mean ± SD)	GAF (mean ± SD)	EAS-T (mean ± SD)	EAS-pc (mean ± SD)	EAS-fc (mean ± SD)	EAS-al (mean ± SD)	SERS (mean ± SD)	S-QOL (mean ± SD)	BIS (mean ± SD)
W0	55.6 ± 16.7	41.9 ± 9.1	37.1 ± 15.1	6.00 ± 3.6	8.6 ± 4.9	10.2 ± 2.7	84.1 ± 14.7	119.9 ± 29.3	9.7 ± 4.5
W12	44.7 ± 8.2	49.4 ± 10.2	29.1 ± 13.5	3.9 ± 4.0	5.5 ± 3.8	8.5 ± 4.3	87.2 ± 10.8	134.7 ± 30.9	9.8 ± 5.1
Pcorr	3.5⋅10^−4^	6⋅10^−3^	0.04	0.02	0.04	0.02	0.26	0.25	0.39

*BPRS, Brief Psychiatric Rating Scale; GAF, global Assessment Functioning; EAS, Evaluation of Autonomy Scale; EAS-T, total score; pc, personal care; fc, financial control; al, affective life; S-QOL, scale for quality of life assessment; BIS, Birchwood Insight Scale*.

### Clinical and Psychosocial Evaluations between W0 and W12

Patients clinically significantly improved at W12 comparatively to W0 for BPRS scores (*p* < 0.001), as well as for GAF scores (*p* < 0.01) (see Table 2).

For psychosocial evaluations, there was a significant improvement for the EAS-Total score (*p* < 0.01); when going through the different dimensions, the W12-W0 change for the EAS-personal care dimension was significant (*p* < 0.05) as well as the EAS-affective life (*p* < 0.05) and financial control (*p* < 0.05). For the S-QOL questionnaire, as well as the Insight short form questionnaire, no difference was found for the total scores between W12 and W0. Concerning the SERS, no significant difference was found either between W12 and W0 – and it was not either for positive or negative scores of the SERS.

### Pre/Post Comparisons in Neuropsychological Assessment

– *Attention, visual scanning and speed processing (See* Table 3*):* D2 cancelation test: the KL score was significantly different at W12 comparatively to W0 (*p* < 0.05), and so was the GZ-F score (*p* < 0.05). However, there was no significant change for the GZ score (*p* = 0.22), or for the F% score (*p* = 0.19). For the Code subtest of the WAIS-III (32) assessed at W0 and W12, the change for the total score was significant (*p* < 0.05), as well as for the Copy-Code (*p* < 0.05).
– *Verbal and Visual Working memory:* When considering the standard scores of the Digit Span, there was a very significant difference when comparing W12 to W0 (*p* < 0.01), but any difference appeared for the forward span (*p* = 0.25) and the backward span (*p* = 0.14). No significant difference was found for the standard score of the visuo-spatial span subtest (*p* = 0.09) or for the forward span (*p* = 0.11), but a significative difference was found for the backward span (*p* < 0.05).
– *Verbal learning:* no change was reported for the Grober and Buschke test (1987) whatever the score (*p* > 0.10).
– *Executive functioning:* no change was observed either for the score of sequence, time of planning, time of execution, or Number of errors for the Zoo map 1 and 2 test (*p* > 0.05), nor for the action programs (*p* = 0.21) of the BADS.
– *Visuo-spatial organization:* the RCFT revealed no significant change, either for time (*p* = 0.37), or for total scores (*p* = 0.10).
– *Retrospective and prospective memory:* for the VR Retrospective memory Test with binding apprehension, there were significant differences at W12 compared to W0, for the “Where” egocentric recognition scores (*p* ≤ 0.05), and the “When” recognition score (*p* < 0.05). The difference was not significant for the “What “recognition (*p* = 0.09).

**Table 3:** **Cognitive assessments at W0 and W12**.

*N* = 7			W0	W12	*P*-value
D2 cancelation test	KL		130.57 ± 32.89	149.00 ± 26.70	0.02
	GZ		362.14 ± 68.55	378.29 ± 76.02	0.22
	F%		5.97 ± 4.56	3.91 ± 3.83	0.19
	GZ-F		338.71 ± 71.34	368.29 ± 72.45	0.02
WAIS:code	Total		5.57 ± 2.57	7.14 ± 3.18	0.03
	Copy		89.71 ± 23.35	106.14 ± 22.99	0.04
WAIS: Digit Span	Sd score		6.71 ± 1.70	8.57 ± 1.72	9.10^-3^
	Forward span		5.71 ± 0.95	5.42 ± 0.79	0.25
	Backward span		3.43 ± 0.54	3.86 ± 0.90	0.14
WAIS: Visuo-spatial span	Sd score		9.57 ± 1.51	10.71 ± 2.21	0.09
	Forward span		5.86 ± 0.69	6.29 ± 0.95	0.11
	Backward Span		5.00 ± 0.82	5.86 ± 0.38	0.047
Verbal learning test	Immediate recall (/16)		15.14 ± 1.07	14.57 ± 1.27	0.17
	Total free recall (/48)		26.14 ± 7.40	26.71 ± 5.91	0.34
	Total total recall (/48)		42.71 ± 7.54	43.71 ± 4.64	0.34
	Free delayed recall (/16)		9.57 ± 3.51	9.71 ± 3.2	0.45
	Total Delayed recall (/16)		14.57 ± 2.51	14.00 ± 2.24	0.17
	Recognition (/16)		15.86 ± 0.38	16.00 ± 0.00	
Zoo map 1	Score of sequence		5.00 ± 3.79	4.29 ± 3.19	0.39
	Time of planning		139.29 ± 124.76	155.57 ± 148.67	0.31
	Time of execution		237.00 ± 137.82	303.71 ± 146.83	0.19
	Number of errors		2.57 ± 4.32	1.14 ± 1.35	0.09
Zoo map 2	Score of sequence		8.00 ± 0.00	8.00 ± 0.00	
	Time of planning		18.00 ± 31.41	39.43 ± 34.08	0.23
	Time of execution		92.86 ± 42.93	148.00 ± 102.47	0.12
	Number of errors		0.29 ± 0.76	0.29 ± 0.76	
BADS			3.67 ± 0.82	3.00 ± 1.83	0.17
RCFT	Time score		194.43 ± 60.32	192.57 ± 69.58	0.37
	Total score		32.57 ± 1.99	31.86 ± 1.68	0.10

***N* = 6**

Retrospective memory VR task	«What» RS		0.51 ± 0.10	0.63 ± 0.18	0.09
	«Where egocentric» RS		0.45 ± 0.19	0.65 ± 0.19	0.01
	«When» RS		0.29 ± 0.22	0.62 ± 13	0.02
Prospective memory VR task	Event-based	Learning before navigation	0.92 ± 0.04	0.97 ± 0.06	0.23
		Stop in correct place	0.74 ± 0.19	0.82 ± 0.18	0.03
		Performing correct action	0.35 ± 0.40	0.62 ± 0.48	0.03
		Cued recall after navigation	0.90 ± 0.16	0.97 ± 0.06	0.21
	Time-based	Learning before navigation	0.83 ± 0.20	0.79 ± 0.24	0.35
		Stop in correct time	0.16 ± 0.40	0.25 ± 0.41	
		Performing correct action	0.16 ± 0.40	0.25 ± 0.41	
		Cued recall after navigation	0.70 ± 0.24	0.67 ± 0.40	0.39

*WAIS, Wechsler Adult Intelligence Scale; Sd, Standard score; RCFT, Rey–Osterrieth Complex Figure Test; RS, recognition score*.

*Time is given in seconds. Retrospective and prospective memory scores are expressed in terms of ratio*.

Concerning the VR Prospective memory test, event-based component was improved during the navigation unlike time-based component. Participants appeared to be more efficient after remediation, stopping at the correct place (*p* = 0.05), and performing correct actions (*p* < 0.05). There was no difference in learning action–intentions before navigation and cued recall after the navigation.

### Qualitative Results

At week 12: during the assessment, the clinicians gathered the qualitative opinions of the participants. All of them reported a good tolerance. After the program they noticed: for six participants a better organization, for three patients a gain for planning, two reported an enrichment of relatedness, and more visits done outside their home, for four participants a gain in self-confidence, for five participants more awareness of their own difficulties; for three participants a better rhythm in life, four noticed an effort to search for work or professional training, lastly 3/8 participants requested more therapies, especially a cognitive remediation program or a group for social cognition. Also, they mentioned having done more concrete things in their everyday life, with less stress (e.g., completing administrative files for work employment, opening and sorting out post mails, less mess at home, etc.). One participant went to the employment agency searching for a job. Three participants aimed to return to work, or decided to begin training. One participant wrote a paper in a scientific review to talk about his distressful experience when he was hospitalized and reported the fruitful benefit of the combination of CR and therapeutic activities (37).

## Discussion

In this study, we proposed to chronic patients with schizophrenia, institutionalized for years in a daycare therapeutic activity center, a VR program focused on cognition, mainly visuo-spatial abilities and planning. Our first hypothesis was that this VR environment improves patients’ cognitive abilities, prospective memory, and planning. Furthermore, we hypothesized a clinical benefit and an improvement in social functioning compared to their functional status before the program.

Following our first hypothesis, we observed a significant improvement in the KL and GZ-F sub-scores of the D2 cancelation test. Also, there was significant improvement in the WAIS-Code and Copy-Code. The need to navigate, to develop a good visual orienting in the virtual town, and to establish good landmarks all along the itinerary required an ability to focus on details of the virtual environment (38). Furthermore, patients were instructed to discriminate the streets correctly and rapidly, to take quick decisions to find their way around, and to keep in mind details in the streets, shops, or avenues they had already seen. Lastly, the task they had to perform (shopping in the different shops, action planning, etc.) required a strict attention to the game. On the other hand, the manipulation of the joystick requesting the manual motricity, that could explain the improvement of the motor speed processing. Finally, the standard score of the Digit span was also significantly improved after the program, indicating a greater regularity in verbal working memory performance. Interestingly, we found improvement in backward visual-spatial span, indicating a special enhancement of central executive of visuo-spatial working memory. Once again the richness of details to be kept in mind, as well as the multitasking aspect of this virtual game stimulated visuo-spatial working memory (for example, not forgetting to turn left after the grocery, paying attention to time, buying some bread without forgetting to pick up a friend at the railway station, etc.). In the VR retrospective memory test, significant improvements were found at the end of the program for the “Where” egocentric recognition scores as well as the “When” recognition scores. This result is particularly interesting because schizophrenia is related to a deficit in retrieving contextual information and in binding the different features of an event in memory (39). In the VR prospective memory, the recall of event-based actions during the navigation recall task also improved. The VR program seems to benefit maintenance of action–intentions, detection of prospective event-based cues and intention execution that are generally deficient in schizophrenia (40). Nevertheless, there was no improvement regarding the recall of time-based actions. It could be relevant to augment activities related to time processing (respect of duration or hour) during the VR program. Although encouraging, these positive results have to be cautiously interpreted, as they could reflect performances due to a practice effect. Indeed, the versions used before and after the program were the same. It could be methodologically more rigorous to assess patients with equivalent but different versions of the retrospective and prospective memory tests before/after the program.

Clinically, the BPRS and the GAF scales were very significantly improved. These benefits corroborated the qualitative reports done by the patients. These improvements were noticeable for symptoms as well for qualitative reports of everyday functioning. However, when examining carefully the GAF scores, although there was an average significant improvement, there also was a large variability of the scores after the program. Nevertheless, when examining the BPRS scores, the substantial improvement represented 19.6% of the total scores. This percentage of improvement was similar to what is generally admitted for a pharmacologically active treatment. Hence, this VR program could have a positive, noticeable clinical incidence. However, this result must be tempered by several limitations: the rater was not blind to the treatment, the size of the sample was rather small, and lastly there was no control group to allow a strict comparison versus therapy as usual or versus another treatment-arm. Concerning psychosocial evaluations, there was a significant improvement in the global score of the EAS, with a better personal presentation, a gain in autonomy and improved affective and social relatedness. However, neither the self-esteem, nor the quality of life, nor the insight questionnaires were significantly improved. This has to be considered with a larger group of subjects, or maybe over a longer period of time, as the program only lasted 3 months. This interval is rather short to raise a substantial change for either insight or self-esteem.

These outpatients suffering from schizophrenia or schizoaffective disorder were all chronic users of daycare institutions. They were unemployed, going routinely for years to weekly therapeutic activities, with limited autonomy and complaining of stress and difficulties in their everyday functioning. Two of them were even taking clozapine, indicating a severe form of the disease. The only positive common point when the program began was that they were willing to evolve in their lives. At the end of the program, all reported benefits, with several qualitative points that seemed crucial benefits in everyday functioning: better rhythm in their life, less stress, gain of time, better ability to plan, many things to do at home, concrete things done in their life. The treatment was perfectly well tolerated. The two dropouts happened after the first sessions of the program, probably due to an insufficient motivation to continue. One has to note, however, that a patient did not want to come for the neuropsychological part of the post-program evaluation. This might be due to the challenge this evaluation might represent for some of them. It could be important for the future to put the participants at ease concerning the tests, explaining them that these results will have no incidence for their outcome, treatment, or way of care.

Disconfirming our initial hypothesis, there was no improvement in standard tests of verbal learning and planning. This could be accounted for by several facts: first, the limited power of the tests, due to the small sample. Second, one can notice that the scores obtained by the patients at W0 were high, reaching maybe a ceiling effect. Third, we selected for planning the zoo map test and action planning of the BADS. The zoo map test assesses planning abilities to set up an action plan to solve a problem, define a route for visiting some enclosures in a zoo. This exercise is far from mimicking daily life. Indeed, the participants showed difficulties perceiving the link with the planning abilities required to the everyday life. In addition, the action programs and the zoo tests were not sensitive enough to detect slight variation of performances. Noticeably, ceiling effect was found at W0 for all participants except one at the program action of the BADS, even while participants complained planning difficulties in everyday life activities. However, at W12, patients obtained longer time of planning and execution with the same number of errors, at the Zoo map test, when compared to W0. To explain this discrepancy, we could argue that many participants were penalized, not because of planning disabilities but for taking more time to plan their route. In addition, the action planning and the zoo tests do not provide a sufficiently sensitive quotation that could detect slight variations in performances. It could be better for the next groups to select other tests, such as the modified commission test (41), to obtain a more ecological assessment. In this test, participants have to perform a number of tasks in a neighborhood, taking into account time, load carrying and optimal distance constraints. In this line, the prospective VR test was able to detect improvement in maintenance and execution of action–intentions that are skills related to planning. It will also be interesting to complete the executive functioning assessment by adding inhibition and flexibility tests. Indeed, the VR program involves many steps with quick decisions to take, and when participants are planning different actions, they have to establish priorities to define their schedule. Hence, there could be automatic actions to inhibit in order to be more efficient in action planning, and also more flexible.

When considering all the positive comments the participants reported, this VR program is undoubtedly an asset for our Center for Cognitive Remediation and Rehabilitation. Despite the small number of patients who participated in the program, all the patients who completed it successfully improved, clinically and functionally. Although not demonstrating a substantial neuropsychological improvement in planning, patients showed attention, working memory, prospective, and retrospective memory benefits. Therefore, our initial hypotheses are only partially confirmed. However, the perspective for this program has been highly enhanced by these pioneering results. Patients are truly evolving in a rehabilitation pathway. They are asking to complete this experience with a cognitive remediation program, or to participate in a social cognition group in order to keep progressing in social interactions. One participant even decided to write a paper in a scientific review, on his own, to describe his unique experience (37). He related his drastic evolution in life in these terms (text translated into English):

« In this paper I want to describe my latest professional experience. Even if this page of my life had a bad ending issue, it is important for me to evolve in a constructive way for my future and to better apprehend my illness. It is by confronting myself with the world of work that I can improve my self-knowledge and paradoxically accept my handicap (…) With the help of cognitive remediation and sector teams I realized (…) that I had to think positively about the traumatic events that happened at work and to drive my own evolution in a positive and fruitful manner. (…) Now, I know more about my illness and I accept it. I know my limitations and I acquired a form of stability in life. » (37).

### Limitations

One of the most important limitations is what has been already mentioned, namely the limited sample included in the group. The second point was the non-parallel versions of some assessments, mainly the prospective and retrospective VR tasks. Also, the participants had very diverse treatments with large variations in the dosage and compounds delivered. However, this study was assessed with patients who are institutionalized and in ecological clinical conditions, containing therefore an exemplarity of users who could visit daily a daycare therapeutic Parisian center.

## Conclusion

Virtual reality worlds offer great promises for the future. For the next groups, we plan to develop different versions of the VR program to induce more flexibility and to increase the training for patients by discovering different areas and new routes during their navigation, develop the complexity of scripts and maybe introduce interactive avatars. In the near future, we will introduce a whole network of public transportation (metro and bus) to enable discussions in the group around travel itineraries, as well as some unpredictable events to oblige patients to be more flexible in their routine action plans, while navigating in a non-stressful environment. We think that these points can be highly helpful for patients to manage their daily lives.

These fruitful results encourage our teams to promote serious games as real cognitive remediation methods. Hence, with larger samples, we would have opportunities to demonstrate benefits also in planning, executive functioning, or even social cognition. Also, this program could be helpful for patients with schizophrenia with strong disabilities in daily living, who stay years in residential care unit for example, or living in apartments with a strong clinician support. Thanks to this program, we could expect that the supportive action of the clinicians who help patients to gain independence in life will be reduced or will focus on the remaining deficits insufficiently improved by the program. More generally, these new VR worlds open avenue for innovative rehabilitation programs for patients with schizophrenia.

## Author Contributions

IA and PP supervised the project. IA was in charge of the clinical assessment. LB-H and ZP were in charge of the neuropsychological assessment and the program sessions. CD was in charge of the program sessions. EO was the engineer who conceived the virtual reality program. MS collected the data and was in charge of the statistical analysis. M-OK and all the participants already mentioned were involved in the redaction of the manuscript.

## Conflict of Interest Statement

The authors declare that the research was conducted in the absence of any commercial or financial relationships that could be construed as a potential conflict of interest.
